# Network Representation of T-Cell Repertoire— A Novel Tool to Analyze Immune Response to Cancer Formation

**DOI:** 10.3389/fimmu.2018.02913

**Published:** 2018-12-11

**Authors:** Avner Priel, Miri Gordin, Hagit Philip, Alona Zilberberg, Sol Efroni

**Affiliations:** The Mina and Everard Goodman Faculty of Life Sciences, Bar-Ilan University, Ramat Gan, Israel

**Keywords:** T cells, T cell repertoire, network analysis, graph theory, machine learning, breast cancer, repertoire sequencing, HER2

## Abstract

The T cell repertoire potentially presents complexity compatible, or greater than, that of the human brain. T cell based immune response is involved with practically every part of human physiology, and high-throughput biology needed to follow the T-cell repertoire has made great leaps with the advent of massive parallel sequencing [1]. Nevertheless, tools to handle and observe the dynamics of this complexity have only recently started to emerge [e.g., 2, 3, 4] in parallel with sequencing technologies. Here, we present a network-based view of the dynamics of the T cell repertoire, during the course of mammary tumors development in a mouse model. The transition from the T cell receptor as a feature, to network-based clustering, followed by network-based temporal analyses, provides novel insights to the workings of the system and provides novel tools to observe cancer progression via the perspective of the immune system. The crux of the approach here is at the network-motivated clustering. The purpose of the clustering step is not merely data reduction and exposing structures, but rather to detect hubs, or attractors, within the T cell receptor repertoire that might shed light on the behavior of the immune system as a dynamic network. The **Clone-Attractor** is in fact an extension of the clone concept, i.e., instead of looking at particular clones we observe the extended clonal network by assigning clusters to graph nodes and edges to adjacent clusters (editing distance metric). Viewing the system as dynamical brings to the fore the notion of an attractors landscape, hence the possibility to chart this space and map the sample state at a given time to a vector in this large space. Based on this representation we applied two different methods to demonstrate its effectiveness in identifying changes in the repertoire that correlate with changes in the phenotype: (1) network analysis of the TCR repertoire in which two measures were calculated and demonstrated the ability to differentiate control from transgenic samples, and, (2) machine learning classifier capable of both stratifying control and trangenic samples, as well as to stratify pre-cancer and cancer samples.

## 1. Introduction

The way by which the immune system deals with complexity of signals, is by building a complex regulation system through its arsenal of tools. This regulation system relies on the ability of T cells and of B cells to present and to communicate through a set of highly variable receptors. In T cells, these receptor are called T cell receptors (TCRs), and their sequence complexity is achieved through a delicate recombination mechanism (5) of T cell DNA. As the sequences determining these recombined regions are unique to each T cell clone (mean length around 13-aa), and since they are relatively short, the recent progress in genome sequencing has made it possible to sequence millions of T cells in parallel, for their TCR type, thereby determining the collection of TCRs from those T cell. This collection has been termed the T Cell Repertoire.

The interaction between T cells and tumor cells during tumor progression is the subject of extensive study. Further, Immunotherapy, which over the past few years have been heralded as a great hope in the fight against cancer, relies on the ability to revert tumor progression, by encouraging some T cells to revert from a previous state of tolerance. In some cases, the immune system is able to eliminate tumors before they become uncontrollable. The role of presentation of tumor specific antigens, Neoantigens, is rapidly taking center stage in such immunotherapy research and treatment, with recent major progress in the clinic (6) pushing the field forward. The mirror image of these neoantigens lies in the immunological repertoire. An ability to respond to antigens is an ability coded into the T cell repertoire. The ability to account for the dynamics of the T cell repertoire is therefore critical to our understanding of immune response to tumor cells.

High-throughput biology, needed to follow the T-cell repertoire, has made great leaps with the advent of massive parallel sequencing (1). Nevertheless, tools to handle and observe the dynamics of this complexity have only recently started to emerge [e.g., 2–4, 7, 8] in parallel with sequencing technologies. Collectively, the sequencing step provides the CDR3 (and possibly flanking regions, with some longer-read technologies) for each of the collected cells. The outcome table, often describing millions of cells, indicates involved clones and is referred to as the Repertoire.

The computational study of T-cell repertoires is challenging due to the complexity of the high-dimensional receptors sequences landscape, as well as its time dependency. Several methods for the computational and statistical analysis of large-scale rep-seq data have been developed to resolve its complexity, and less so its dynamics, and to gain insight into the mechanisms controlling the immune system behavior under various conditions. We mention here, and use later, two major approaches: (1) Network-based analysis, in which clones are associated with vertices of the graph, and edges represent some distance measure between pairs of clones, and (2) Machine learning techniques to relate physiological conditions to a state vector composed of the magnitude of particular clones. In Bashford-Rogers et al. (9) BCR sequences were organized into networks which demonstrated that differences in network connectivity may distinguish between repertoires of healthy individuals from those with Chronic Lymphocytic Leukemia, and possibly other clonal blood disorders. They used measures defined by the Gini Index and cluster sizes. Madi et al. (10) applied network analysis of TCR sequencing data to show that substantial numbers of public CDR3-TCRβ are identical in mice and humans. They further used annotated TCR sequences associated with self-specificities such as autoimmunity and cancer, to demonstrate a link to network clusters.

Greif et al. (11) applied machine learning to develop an SVM-classifier for separating private from public TCR sequences. Their machine is reported to achieve 80% prediction accuracy of public and private status in humans and mice, and was sufficiently robust for public clone prediction across individuals and studies using different library preparation and sequencing protocols. In Ostmeyer et al. (12) the authors developed a statistical classifier to diagnose individuals with multiple sclerosis. Their method includes feature selection step based on snippets derived from the BCR sequences that are converted into a set of chemical features using Atchley factors. Those features are combined using logistic regression function whose weights are trained. The outcome is further transformed to a single score (probability) used for diagnosis.

In Miho et al. (13) a computational method is proposed to overcome the hurdle posed by the amount of unique sequences [$O\left(10^{5}\right)$ and higher]. The resulting sparse distance matrix is then used to assess global and local properties of the network over individuals, and at the local (clonal) level. Of interest to our study is the redundancy found in the repertoire space of sequences.

In the following we propose to view the immune repertoire dynamics as a nonlinear dynamical system [see e.g., (14)] whose attractor landscape is characterized by the clusters of similar sequences, hence denoted as **Clone-Attractor** (CA). This representation assumes an inherent robustness, or redundancy, in the repertoire. By this we mean that a cluster of highly similar sequences may be viewed as an attractor, where larger clusters have larger basin of attraction. Sequences belonging to the same cluster-attractor may be relevant to a specific antigen. This representation is used to demonstrate the differences between experiment and transgenic mice via two approaches: (1) network analysis of the TCR repertoire and, (2) machine learning study aim at developing a classification tool to separate experiment from transgenic, as well as the status of a sample as pre-cancer vs. cancer.

## 2. Methods

Temporal TCR repertoire analysis poses a unique problem, as the number of different sequences is very large and (unlike, e.g., gene expression data) changes over time, whereas the amount of samples available in each experiment is relatively small. Since data is collected over several time points, sequences are observed in part of the samples, part of the time, rendering the association of particular clones to complex physiological conditions uniquely challenging. This assertion is even stronger assuming the condition is dominated by multiple clones with possible interactions between their members. We used a cluster-based representation of the repertoire to tackle these difficulties. This representation further makes the analyses more robust. This robustness is gained by treating each cluster as “Clone-Attractor” (CA) whose amplitude is the sum of its members amplitude at each time point.

In the following we describe the clustering algorithm used, followed by a description of two analysis approaches: (1) Graph theoretic measures of the various networks, and (2) Machine learning methods applied to the space of CAs in order to expose a subspace sufficient for classification of control vs. transgenic samples, as well as to stratify pre-cancer and cancer samples.

### 2.1. Experimental Setup, Data Collection and Preprocessing

**Figure 1:**
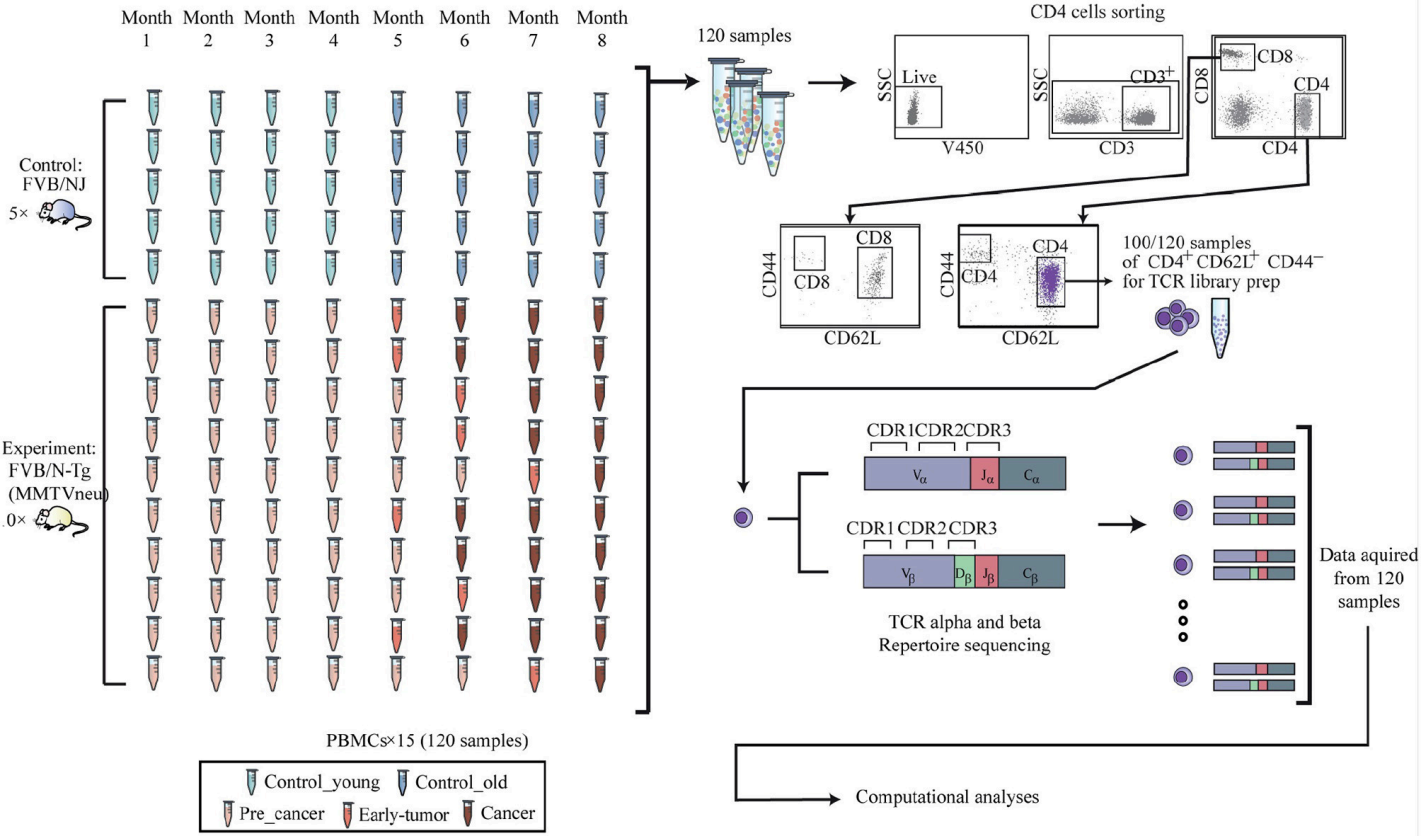
Experimental procedure 120 blood samples were drawn from the retro-orbital sinus of 10 FVB/N-Tg (MMTVneu), a mouse model of HER2 human breast cancer mice, and from 5 FVB/NJ control mice. Over these 8 time points, none of the control mice (blue) developed any tumors. Progress of tumor in the ten transgenic mice is demonstrated using the red colored samples in the figure. The last time point before tumors are shown was defined as pre-cancer and marked light red. From each time point, the peripheral blood mononuclear cells were isolated and stained for flow cytometry. Cells were analyzed and gated for sorting using a FACS ARIA III sorter, and CD4+CD62L+CD44- naive population was separated for RNA extraction and T cell receptor library preparation.

Full details of the data collection and preprocessing are given in Gordin et al. (15). TCR sequencing data, from FASTQ files, has been analyzed using MiXCR (16) to produce CDR3 abundance levels per sample. Table summarizing the number and groups of samples and time points and the number of sequences obtained per sample and time point is given in the **Supplementary Material**. These repertoires were the basis for the network analyses described in the next sub sections. The setup is depicted in Figure 1.

#### 2.1.1. Transgenic Mice

Transgenic Mice expressing the inactivated rat neu (Erbb2) oncogene under the transcriptional control of the mouse mammary tumor virus promoter were purchased from Jackson Laboratories [FVB/N-Tg(MMTVneu) 202 Mul/J]. The female mice of this strain represent a mouse model of mammary tumor in humans, model of HER2/ Erbb2 / Neu human breast cancer (17). FVB/NJ strain with the same genetic background as the transgenic mice, serve as a non-transgenic control mouse that does not develop tumors. Mice were housed in accordance with all applicable laws and regulations following approval by the responsible animal care and ethical committee, under specific pathogen-free conditions. Mice were monitored by palpitation for tumor development monthly for up to 9 months.

#### 2.1.2. Antibody Staining and Cell Sorting

Blood was sampled from the retro-orbital sinus of 15 mice once per month for 8 time points (total of 120 samples). Mononuclear cells from the peripheral blood was isolated by density gradient centrifugation using Ficoll (Ficoll PaqueTM plus, GE Health Care), Single cell suspensions were prepared from thymus and spleen that were removed from each mouse at the end of the experiment. For cell sorting, cells were stained with the following fluorescently labeled monoclonal antibodies: anti-CD4 Pacific Blue (BD), anti-CD25 PE (eBioscience), anti-CD44 APC (BD) and anti-CD62L PE-Cy7 (eBioscience) and viability using the Fixable Viability stain 450 (BD Horizon). Cell sorting was performed using FACS ARIA III sorter. CD4+ D44loCD62Lhi were sorted as naive T cells. After sorting, cells were pelleted and resuspended with 300μ*l* of RNA protect cell reagent (Qiagen). Cells were stored at minus 80°*C* until RNA extraction. RNA was purified from RNAprotect-stabilized cells using the RNeasy Plus Mini Kit. After RNA extraction, samples were run on TapeStation to estimate quality.

#### 2.1.3. High-Throughput Sequencing of the T Cell Repertoire

The method for high-throughput sequencing of the T cell repertoire was performed as previously described in Di Niro et al. (18) and Tsioris et al. (19). Briefly, RNA was reverse-transcribed into cDNA using a biotinylated oligo dT primer. An adaptor sequence was added to the 3' end of all cDNA, which contains the Illumina P7 universal priming site and a 17-nucleotide unique molecular identifier (UMI). Products were purified using streptavidin-coated magnetic beads followed by a primary PCR reaction using a pool of primers targeting the TCRα and TCRβ regions, as well as a sample-indexed Illumina P7C7 primer. The TCR-specific primers contained tails corresponding to the Illumina P5 sequence. PCR products were then purified using AMPure XP beads. A secondary PCR was performed to add the Illumina C5 clustering sequence to the end of the molecule containing the constant region. The number of secondary PCR cycles was tailored to each sample to avoid entering plateau phase, as judged by a prior quantitative PCR analysis. Final products were purified, quantified with Agilent Tapestation and pooled in equimolar proportions, followed by high-throughput paired-end sequencing on the Illumina MiSeq platform. For sequencing, the Illumina 600 cycle kit was used with the modifications that 325 cycles was used for read 1, 6 cycles for the index reads, 300 cycles for read 2 and a 20% PhiX spike-in to increase sequence diversity.

### 2.2. Clustering Algorithm

The clustering method we used, roughly follows the UClust (20) algorithm with some modifications. Its purpose is twofold: (1) data reduction, i.e., mapping the very large space of unique sequences to the space of representative clusters, 2–3 orders of magnitudes smaller, and (2) reducing the inherent fluctuations in the data, assuming very similar TCR-sequences are associated. In addition, we naturally minimize the occurrence of missing values, a phenomenon in which many algorithms struggle [e.g., see (21, 22)], since the activity of each cluster (CA) is now based on several sequences. The graph nodes (or features) are considerably less sensitive to the noise in measuring the single sequences.

The algorithm begins by sorting the sequences according to their length and starting from the smallest. It then iteratively checks for existing cluster to associate the next sequence whose editing distance from the cluster's representative is smaller than a given threshold. The association step is greedy, namely, to the first cluster that meets the constraint. The editing distance used was 'Levenshtein' with parameters [*deletion* = 1.1, *insertion* = 1.1, *substitution* = 1.9]. The association threshold was set to λ = 3. This choice of parameters ensures at most 2 deletions/insertions, or 1 substitution plus 1 insertion/deletion with respect to the 'cluster-representative' sequence.

Following is the pseudo-code describing the algorithm. Let us denote the current set of already found clusters by *C* = *c*_1_, *c*_2_, ⋯ , *c*_*k*_, where each cluster's representative is denoted by *Cr* = *cr*_1_, *cr*_2_, ⋯ , *cr*_*k*_. Each *c*_*j*_ is the set of all sequences associated with the j'th cluster.

1. Read one sequence, denoted as $\hat{x}$
2. Calculate similarity measure $S\left(\hat{x},cr_{j}\right)\forall j$, i.e., Levenshtein distance, between the sequence and the j'th cluster representative
3. Find the nearest cluster *c*_*i*_ to $\hat{x}$
  (a) Associate $\hat{x}$ to the most similar cluster *c*_*i*_ if $S\left(\hat{x},cr_{i}\right)\leq \lambda$. Update cluster representative by searching for a new member of the cluster that minimizes the distance from all other members
  (b) If no cluster found, i.e., $S\left(\hat{x},cr_{i}\right)>\lambda \forall i$, create a new cluster *c*_*k*+1_ with representative $cr_{k+1}=\hat{x}$ and add it to the set *C*
4. Repeat the above steps until exhausting all sequences

The algorithm goes over all sequences once, and the number of clusters found depends on the threshold λ defining the “radius” of the CAs, i.e., the ensemble of highly similar sequences. As mentioned, to reduce the complexity of the algorithm, we adopted a greedy strategy in which the current sequence is associated to the first cluster that is found close enough (winner takes all).

### 2.3. Graph Theoretic Analysis

Our temporal data give rise to multiple graphs, each represents a sample at a given time-point. Graphs were generated based on the CAs as nodes, and the distance between the representative sequences of each pair of CA as edges. Nodes with < 10 members (||*CA*_*i*_|| < 10) were eliminated. Edges of distance >8 were eliminated as well. Finally, we kept only CAs that appeared in more than 60% of the time points. So, starting from ~ 360*k* sequences, we obtained ~ 57*k* CAs, from which ~ 550 CAs remained after applying the above filtering process. Nevertheless, those remaining CAs account for ~ 100*k* of all sequences. The above parameters were chosen empirically, taking into consideration both robustness and complexity issues. That is, we opt for taking considerable amount of CA's, however, those CA's should be statistically significant (hence the cutoff at 10 members). In addition, we require them to cover enough time points to ensure they represent a phenomenon and not a sample. The exact parameters' value is less important, and one can vary them to filter more or less CAs. The results shown below are not sensitive to these parameters. We tested various sets of parameters that resulted in an amount of CA's that roughly varies in the range 400 − 1, 000.

To compare the various graphs, we build the following quantities to reflect measures of the graphs (other than visual inspection), which are required for an unbiased comparison of non-trivial and large networks. Many such measures have been developed within the field of graph theoretical analysis [see (23)]. We demonstrate the differences between the control/transgenic groups using two measures, namely, the Betweenness Centrality (BWC) which is a node level measure, and the Molecular Topological Index (MTI) which is a graph level measure.

The molecular topological index originated from the study of graph representation in (mathematical) chemistry (24), and some of its properties can be found in Gutman (25). The MTI is defined by

(1) $$\begin{array}{r} MTI=\overset{n}{\underset{i=1}{\sum }}\overset{n}{\underset{j=1}{\sum }}d_{i}\left(A_{ij}+D_{ij}\right) \end{array}$$

where *n* is the number of vertices of the graph, *d*_*i*_ is the degree vector of the vertices, *A*_*ij*_ are the entries of the adjacency matrix A (*A*_*ij*_ is 1 if vertices i and j are adjacent and 0 otherwise), and D the graph distance matrix, i.e., the number of edges on the shortest path. One of its properties, relevant to our case, is the inverse relation between its value and the graph “branchness.”

The betweenness centrality (one of several centrality measures) is defined as follows:

(2) $$\begin{array}{r} BWC\left(i\right)=\underset{i\neq j\neq k}{\sum }\frac{g_{jk}\left(i\right)}{g_{jk}} \end{array}$$

where *g*_*jk*_ is the total number of shortest paths from node *j* to node *k* and *g*_*jk*_(*i*) is the subset of paths that pass through *i*. The BWC is a measure of accessibility, i.e., the number of times a node is crossed by shortest paths in the graph between pairs of nodes *j* − *k*.

Since the BWC is a node level measure, we basically evaluate its quantity for every graph node. Although we begin the process of building the graph for each sample from the same set of CA's, the effective size of each graph (based on the activity of the nodes/CA's at that time-point) is different. To facilitate the comparison between the graphs, we evaluate a single **global** variable from each vector of $\vec{BWC}$ values, being the sum of all components above some threshold taken as the median of all BWC vectors (*th*_50_). This global variable is in fact the temporal-graph-mean-BWC (since the original number of nodes is the same). It's biological meaning is then: “the average amount of influential CA's.”

(3) $$\begin{array}{r} sBWC=\underset{i}{\sum }BWC_{i},\forall BWC_{i}>th_{50},i=1..n \end{array}$$

We note that the results presented below are not sensitive to the threshold chosen, i.e., other statistical values will work as well.

### 2.4. Machine Learning Methods

While using graph theoretic measures can shed light on global level differences between networks (in our case, of different genetic and/or physiologic origin), the purpose of applying machine learning methods is to identify particular representations that will provide efficient classification results, but, just as important, an efficient geometrical representation. Since the number of data points in our experiments, i.e., samples at different time points, is small in terms of statistical machine learning, especially with respect to the original dimensionality of the data, it is imperative from the generalization point of view to obtain a robust, low-dimensional solution.

#### 2.4.1. Feature Selection

The first step involves feature selection. In our case, the features are the magnitudes of each Clone-Attractor, taken per sample per time point. Since the number of CAs is relatively high, while the number of data points is very small, we first reduced the set of CAs to the subset that is active across samples (> 95% of samples). “Active” in this context means that at least one sequence in the CA is expressed in a sample/time-point. This process resulted in < 100 CAs.

To search this, still very high, feature space we adopted a sequential bottom-up (forward) scheme. The two classes for this step where Control/Transgene for which there were 24/49 data points respectively. The classifier used was SVM with “Gaussian” kernel (26, 27). Instead of starting from choosing among all single features, we trained 2D classifiers on all pairs of CA features. Based on the leave-one-out cross validation (LOOCV) (28), the top-50 pairs were chosen to continue. This process has been repeated for the subsequent iterations until the overall performance converged. At the end of this stage we obtained the best *k* = 50 sets of features for each dimension.

#### 2.4.2. Robust Model Evaluation

One of the major problems in assessing performance of a learning machine based on a very small data set is the robustness of the solution, or the generalization error. Since its impractical to apply the standard statistical learning methodology, i.e., to subdivide the data set into training/validation/test sets, due to its size, we combined the following techniques:

1. Using LOOCV, as described above for the feature selection phase
2. Naive form of ensemble averaging (29) - committee of classifiers trained on different feature subspace
3. Model testing via noisy versions of the original data

Using ensemble averaging of *m* = 10 machines reduced the variance of the combined (meta) classifier, as expected. In order to obtain a more robust evaluation of the model, we generated noisy data sets, each with a higher noise amplitude. Each noisy set has been generated as follows. Let us denote the original set by $\vec{X}$, then the k'th noisy set ${\vec{nX}}^{k}$ is obtained by multiplying the data by random normally distributed variable with variance $\sigma_{k}^{2}$, i.e.,

(4) $$\begin{array}{r} nX_{i}^{k}=X_{i}\left(1+V_{i}^{k}\right)i=1..n,V\sim N\left(0,\sigma_{k}^{2}\right) \end{array}$$

We used noise amplitudes varying in the range [0, …, 0.25].

## 3. Results

### 3.1. Cluster Analysis Results

The fundamental step in our analysis is clustering the T-Cell repertoire sequences and generating “Clone-Attractors” (CAs). Due to the smaller amount of TCRα sequences, and the higher occurrence of time points absent of TCRα sequences, we show results of TCRβ only.

**Figure 2:**
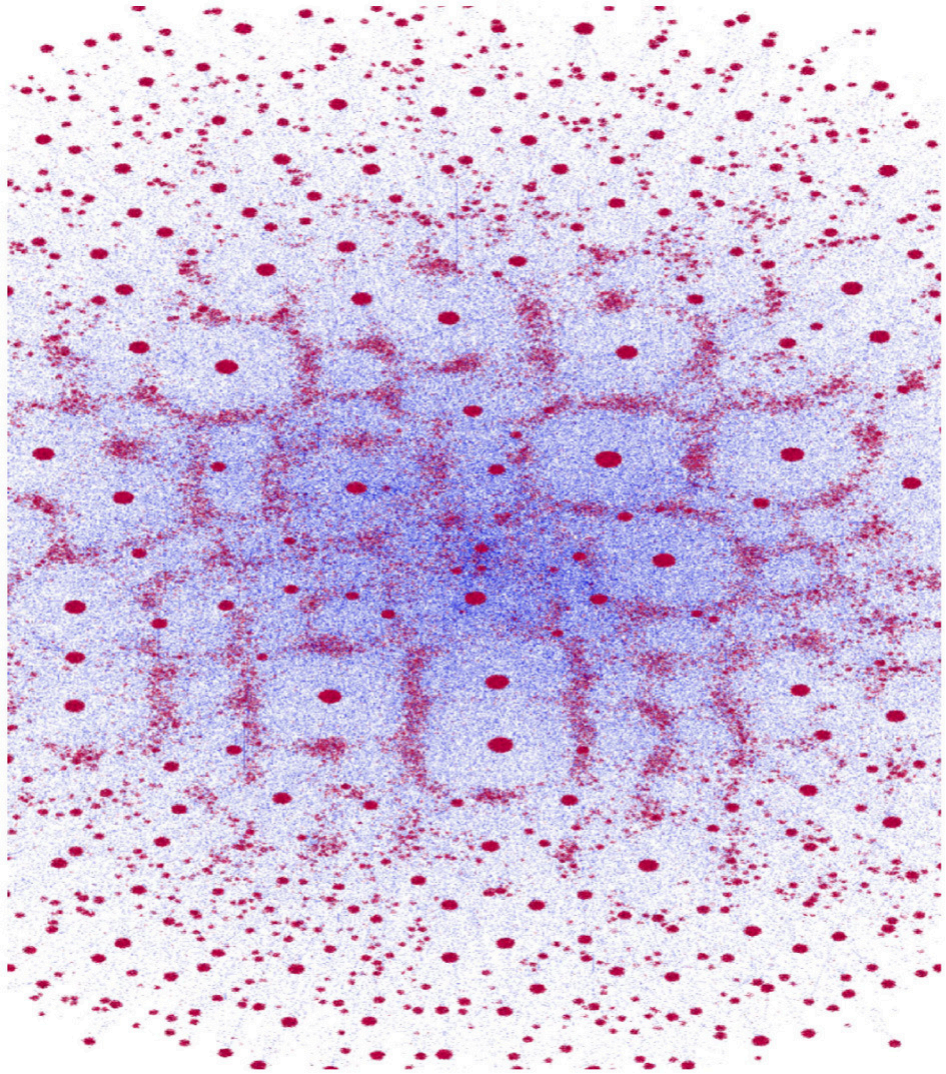
Clone-Attractors network. Red dots represent nodes (CAs) of the graph (size proportional to the number of sequences in the CA), and blue lines are the edges (line width is inversely proportional to the distance.

The original data set obtained comprised of ≈ 360*kTCRβ* sequences. Following the clustering procedure, the number of clusters found was ≈ 57*k*. Figure 2 depicts the network of the CAs obtained from all the sequences. The size of each red circle is proportional to the size of the CA (number of sequences associated) and the blue lines correspond to the graph edges (line width is inversely proportional to the distance between each pair of CAs). The figure has been generated using “Gephi” (30).

**Figure 3:**
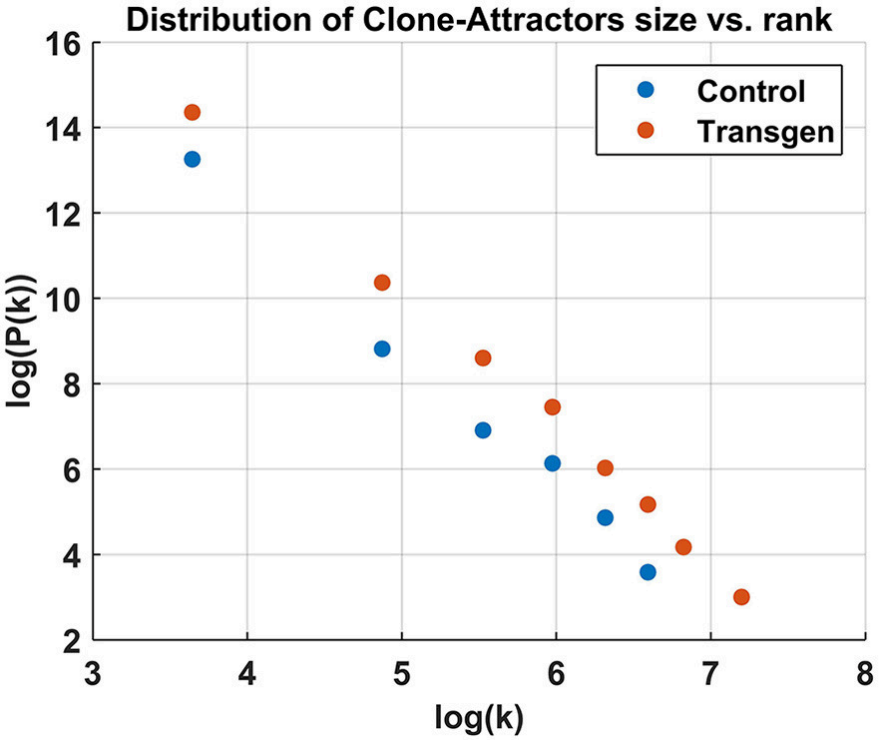
Cluster size distribution in log-log scale, showing a power-law relation. The analysis was done for all the active CAs combined from the Control/Transgenic samples, respectively.

A quick examination of Figure 2 reveals a small number of highly connected CAs (hubs) and numerous more isolated ones. This qualitative observation is verified in Figure 3, where the distribution of cluster sizes is shown to follow a power-law scaling (31),

$$P\left(K\right)\propto {\left(K\right)}^{-\alpha },K=||CA||$$

This result holds for all samples/time-points, with different pre-factors and slightly different power values, where α≈3. This is a strong indication that the network belongs to the class of scale-free networks.

**Figure 4:**
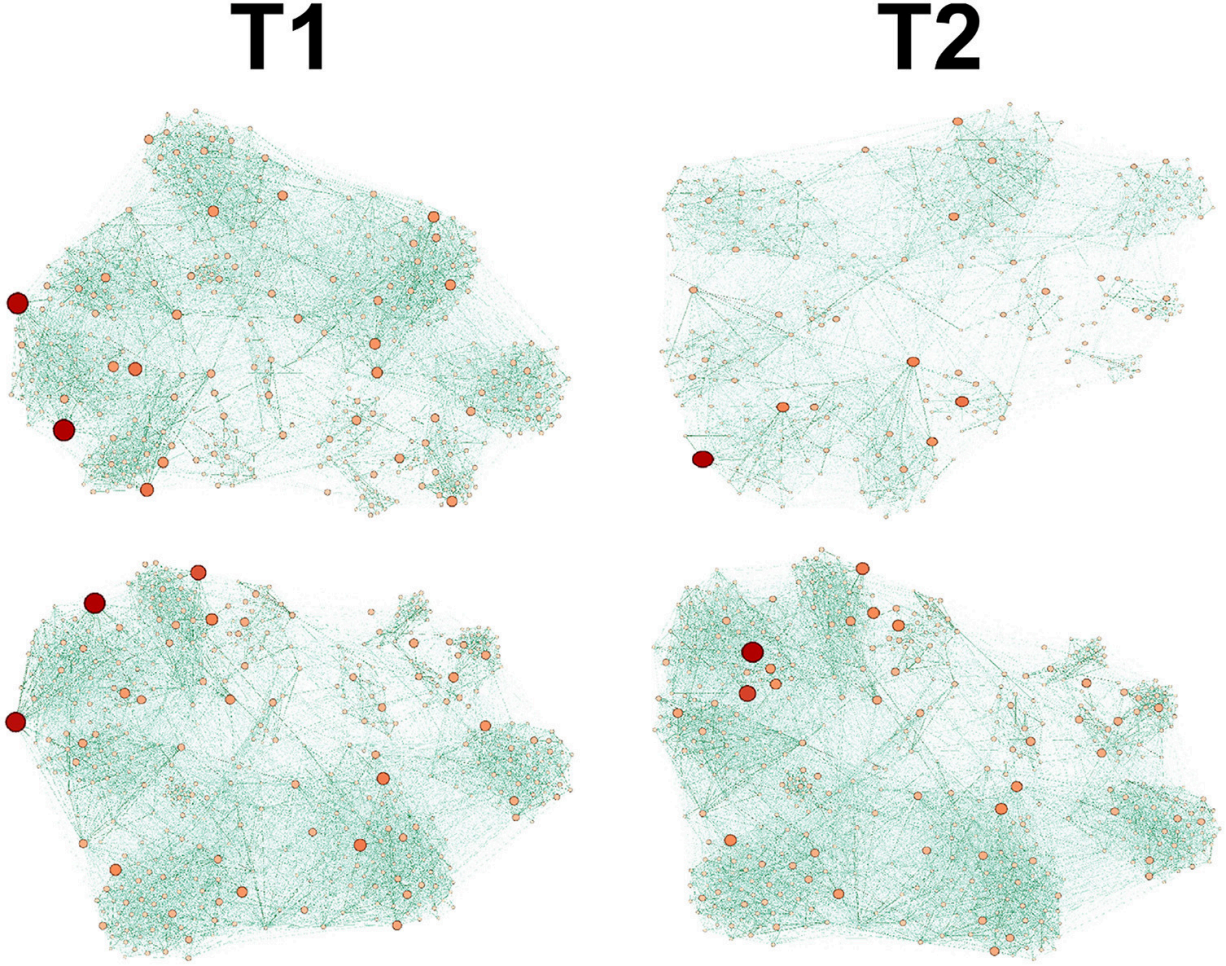
Graph dynamics of Control (up) and Transgenic samples (down), depicted at 2 time points, *T*_1_<*T*_2_. The Transgenic graph remains dense, whereas the Control becomes branched and diluted at later time.

Before we provide results of the graph theoretic analysis, it is useful to see the panel (Figure 4) of example Control vs. Transgenic networks at two time points along the experiment, early/late (denoted *T*_1_/*T*_2_ respectively). It is evident from the figure that while the network of the Control mice becomes more sparse, the network of the Transgenic mice remains densely connected. In the next subsection we elaborate on the quantitative results regarding this behavior.

### 3.2. Graph Theoretic Results

Using the clustering algorithm for all repertoire sequences, resulted in an array of CAs as described in 2.2. Since the TCR repertoire was generated for each sample, control and transgenic, at several time points, we generated multiple graphs from the active CAs from each pair (time ↔ sample). As mentioned above, we filtered the CAs such that the remaining subset contained only those clusters that were found active in most samples/time-points. As mentioned in section 2.3, the filtering process resulted in ~ 550 CAs upon which the results below were obtained, i.e., these CAs were the graph's nodes.

The measures described in 2.3, Betweenness Centrality and Molecular Topological Index, were calculated for each sample/time-point. In the next two figures we present the median value of all time points per sample. In both figures the median and std are presented for each sample, where the std is calculated over time points.

**Figure 5:**
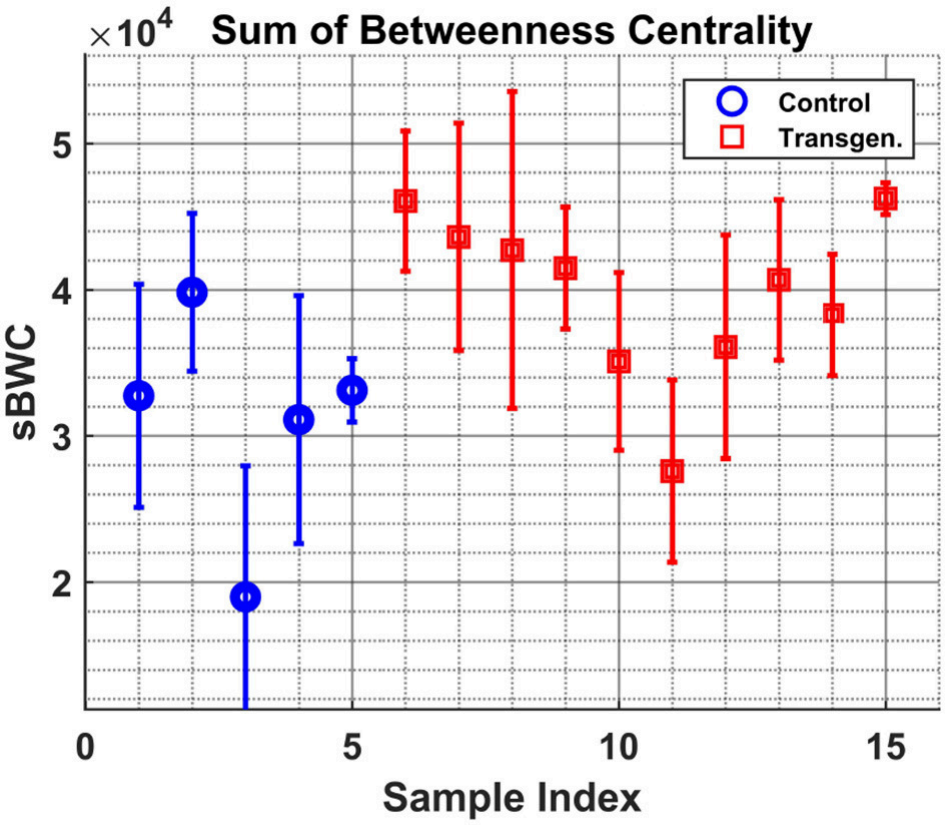
Samples graph Betweenness-Centrality. The sum of BWC of each sample (indices [1–5] - Control, [6–15] - Transgenic) is shown along with the 1σ errorbar.

**Figure 6:**
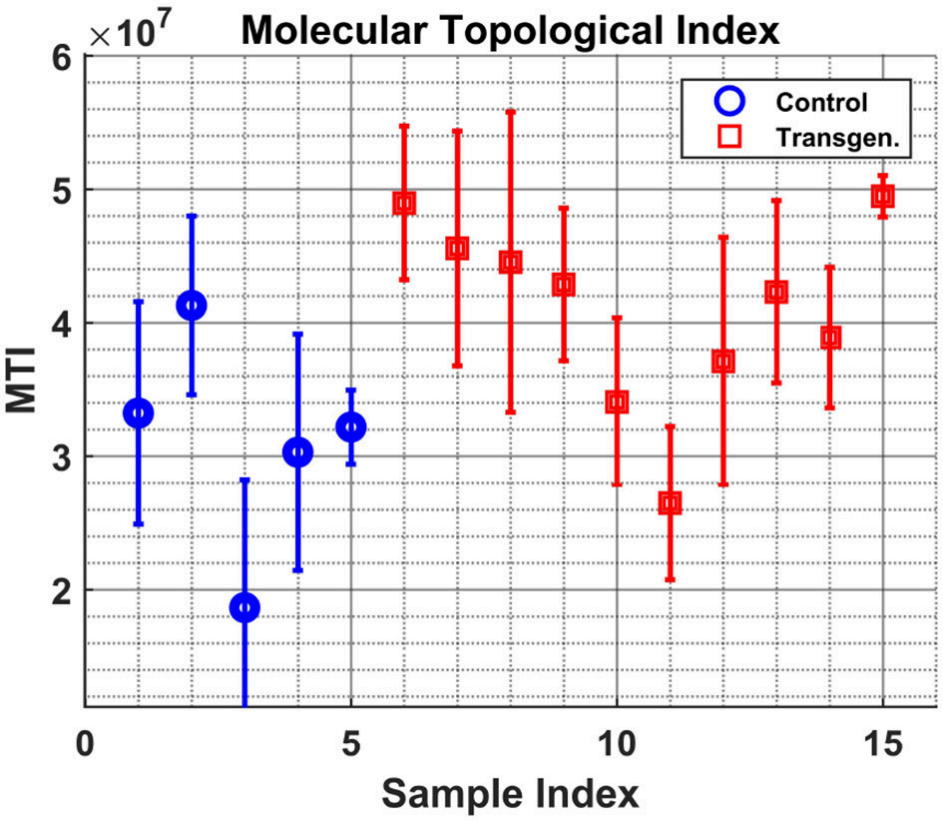
Samples graph Molecular-Topological-Index. Similar to Figure 5, but for the MTI measure.

Figure 5 shows the variable sBWC (Equation 3) averaged over time per each sample. The separation between the two groups is apparent, where 80% success rate was achieved in distinguishing control (4 out of 5) from transgenic sample (8 out of 10). The same result is obtained using the MTI in Figure 6.

It is worth noting that the lower levels of the MTI measure in the control group may be attributed to the graph 'branchness' observed at later times (see Figure 4). Similarly, the lower levels of the sBWC are associated with the decreasing number and amplitude of significant nodes (or hubs), again at later times, in the control group.

### 3.3. Machine Learning Based Classification

As mentioned earlier, the overall data available for analysis (from the 5-control and 10-transgenic mice) consisted of 73 time points, of which 24 from control and 49 from transgenic. Prior to the feature selection process described in 2.4, the data is about hundred dimensional, originated from the CAs.

We applied the machine learning pipeline described in section 2.4 in two stages. First, we applied to classify the Control and Transgen groups. Assuming the first stage is successful, we then used the same pipeline to generate another classification machine to classify the pre-cancer and cancer sub populations within those classified as Transgenic. Indeed, it turns out that the subset of features found in the second stage are mostly different than those found in the first stage. This hierarchical scheme allowed us to separate the two problems and control the learning process, in particular in view of the small size data set at hand.

**Figure 7:**
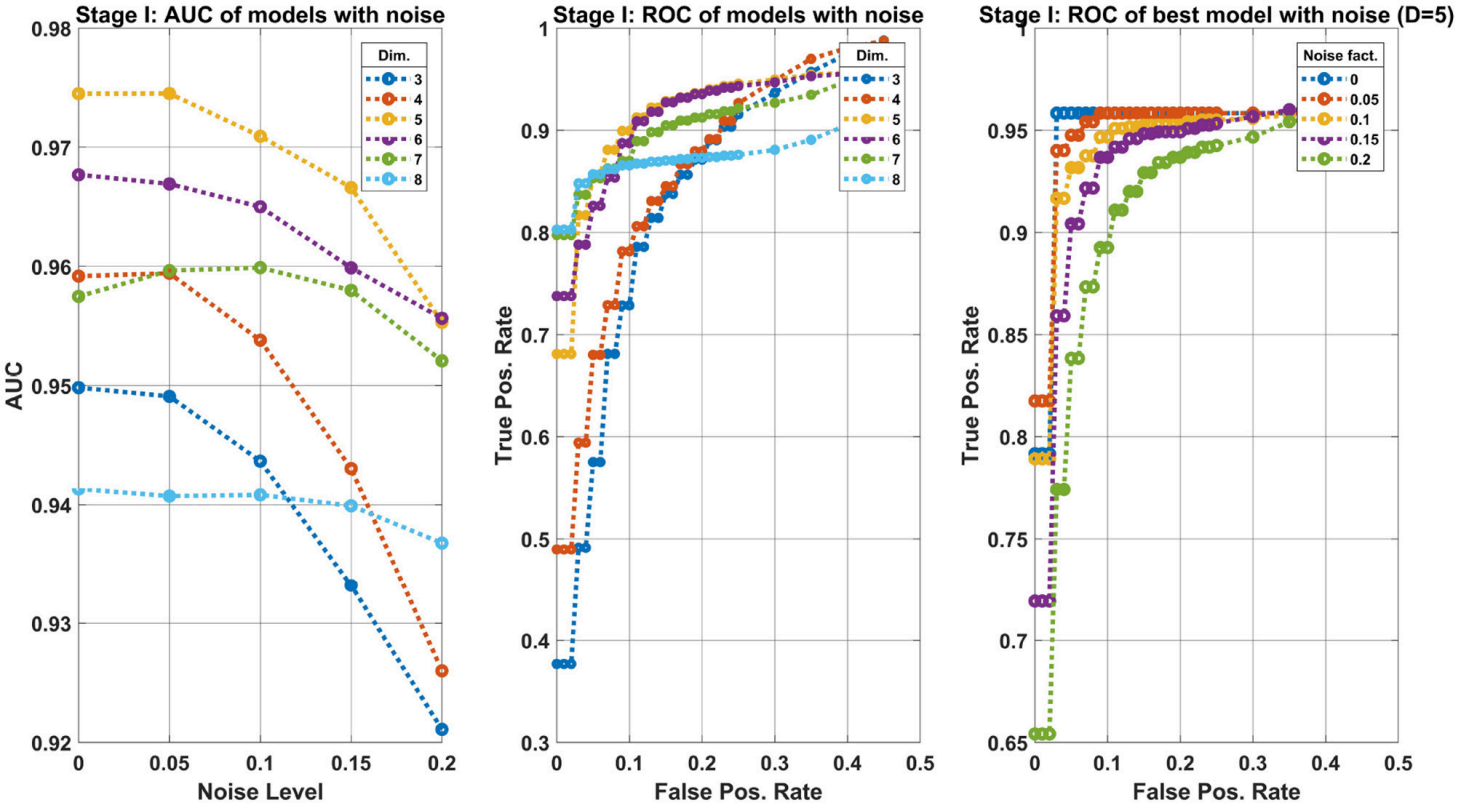
Machine learning results of the first classification stage: separating Control from Transgenic classes. **Left panel**: AUC of models trained on different dimensional features space (see legend) at various noise levels (Equation 4). The 5-dimensional model is preferred. **Middle panel**: ROC of the same models as the left panel, where each point is the average over the noise levels. This curve allows us to prefer the model *D* = 5 assuming our operating point is *FPR* = 0.1. **Right panel**: Zoom in the best model, showing the performance degradation for various levels of noise added to the data.

Figure 7 summarizes the results of the first stage. The left side panel shows the Area-Under-Curve (AUC) of several classifier models trained as described above. Each classifier model (an ensemble of 10 machines of the same input dimension) operates on a different dimensional space, shown are *Dim* = 3, …, 8. The models were tested with various levels of noise amplitudes, ranging *noise* = 0, …, 0.25. The best model, according to the AUC is obtained for *D* = 5. The middle panel shows the Receiver-Operating-Characteristic curve (ROC), i.e., the true positive rate (TPR) vs. the false positive rate (FPR) calculated at various threshold values of the classifier's output. The values of each point are the average over the noise level tested. The models *D* = 5, 6 perform the best, hence we shall take the lower dimensional model. Finally, the graph on the right shows the ROC for the chosen model (*D* = 5) for various noise levels. The robustness of the model is evident by the gradual decrease in performance as a function of the noise. One can set the operating point of the classifier at *FPR* = 0.1 to obtain *TPR*≈0.9. The TPR value is taken at the worst noise level.

Note that the FPR refers to the expected error in the Control group, whereas the TPR refers to the Transgenic group. More specifically, at this operating point, there is a 0.1 probability of misclassifying a Control sample as a Transgenic, and about 0.9 of correctly classifying a Transgenic sample.

**Figure 8:**
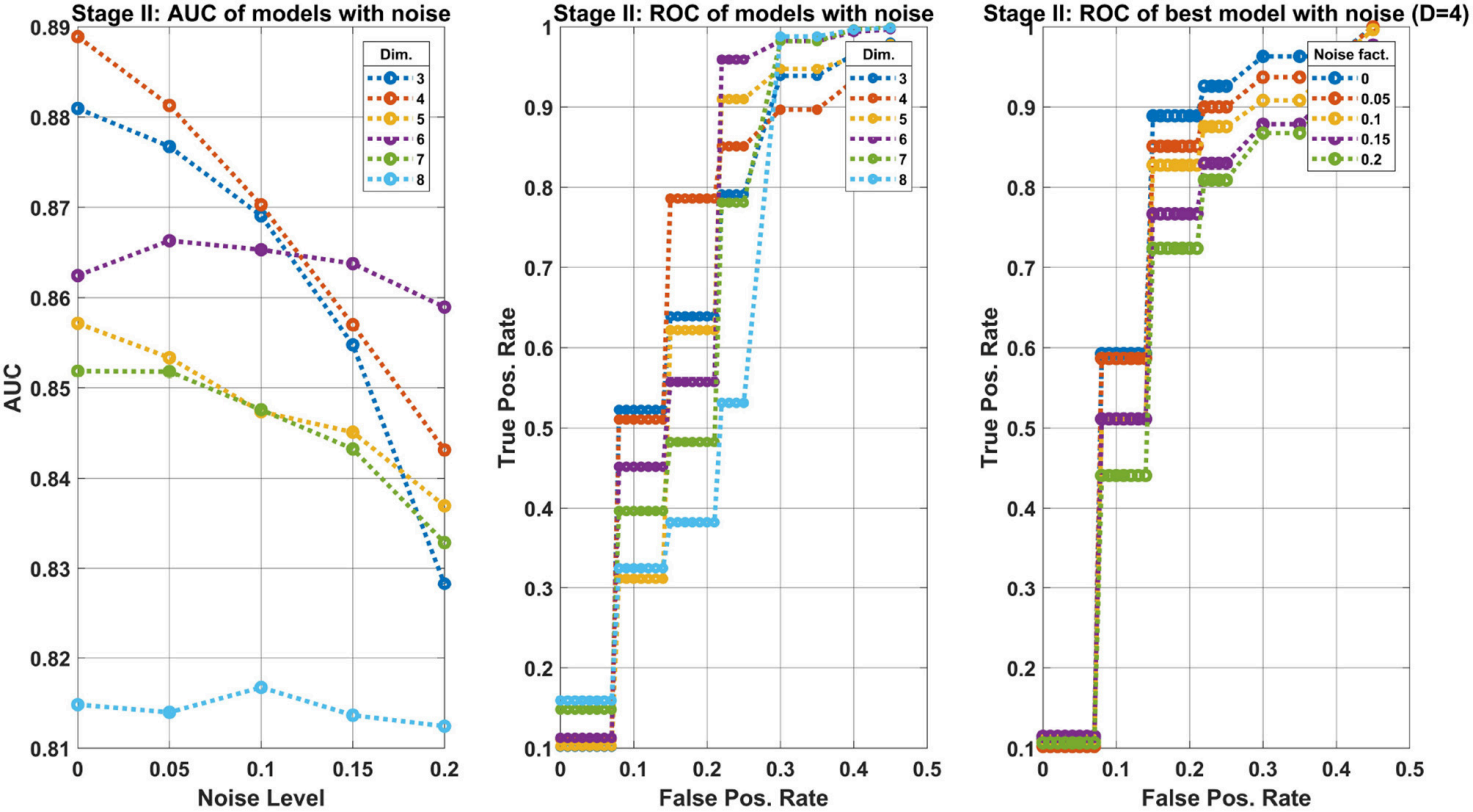
Machine learning results of the second classification stage: separating precancer from cancer classes of the Transgenic group. The structure of the figure are as Figure 7. The main difference between the two stages is the reduced performance observed here. In particular, the best model achieves a *TPR*≈0.8 at an operating point where *FPR*≈0.2.

The results depicted in Figure 8 refer to the second classification stage, i.e., of separating the classes pre-cancer/cancer of the Transgenic group. The details of the three panels in the figure are identical to Figure 7. However, the main conclusion here are that the performance of the best ensemble are reduced with-respect-to the first classification stage. One may expect at *FPR*≈0.2 to obtain *TPR*≈0.8.

As noted, the set of features (CAs) found for the two classification stages are different, indicating that there might be two biological processes involved. Referring to Table S1 in the **Supplementary Material**, the list of sequences denoted as: [1, 2, 10, 13, 17, 23, 26, 44, 48, 60, 68, 71] was found best for stage-1, and the list: [3, 5, 11, 16, 32, 33, 35, 38, 42, 63, 64, 77, 82] was found best for stage-2.

### 3.4. Correlation With Public TCR DataBase

The growing number of availble rep-seq datasets, over multiple phenotypes, enabled the production of curated databases of T-cell receptor (TCR) sequences with associated antigens. One such database is the VDJdb (32) (see project web-page https://vdjdb.cdr3.net), whose primary goal is to facilitate access to existing information on T-cell receptor antigen specificities, i.e., the ability to recognize certain epitopes in a certain MHC contexts.

Out interest in these types of Db's is 2-fold: analyzing the extent of public sequences in private repertoire, and correlating the sequences with our CA representation. The VDJdb currently contains ≈16*k* β− sequences. Analysis of the distance matrix between the VDJdb sequences and our CAs reveals the following interesting results. When taking into account the CAs used for the graphs analysis (≈550, section 2.3), the number of sequences from the VDJdb whose distance (*d*) from any of those CAs is d = 0, 1 amount to 4, 126, respectively. That is, four sequences were identical to CAs representatives, and another 126 differ by a single insertion/deletion from CAs. Of interest is the fact that out of those 126 sequences, 38 are identical to one of the members of the respective CAs.

As for the CAs chosen for the machine learning (ML) study (section 2.4), the number of sequences from the VDJdb whose distance from any of those (ML)CAs is *d* = 0, 1 amount to 2, 64 respectively. Again, out of those 64 sequences, 30 are identical to one of the members of the respective (ML)CAs.

Table 1 presents the set of sequences from the VDJdb that matches CAs found in the ML process described above, i.e., they are among the CAs comprising the feature space upon which the classification machines were built. First 4 sequences matches features found in stage-1 (section 3.3), and the next 4 sequences corresponds to features found in stage-2.

**Table 1:** List of sequences from the VDJdb that matches CAs revealed via the machine learning process.

	**AA sequence**	**Species**	**AG gene**	**AG species**	**IGoR Prob**.
1.	“CASSLGGYEQYF”	“Mus-Musculus”	“PA”	“InfluenzaA”	0.29
2.	“CASSPLGANTGQLYF”	“Mus-Musculus”	“PA”	“InfluenzaA”	0.14
3.	“CASSPGTDTQYF”	“HomoSapiens”	“NP177”	“InfluenzaA”	0.12
4.	“CASSPLTDTQYF”	“HomoSapiens”	“BZLF1”	“EBV”	0.1
5.	“CASSPQTDTQYF”	“HomoSapiens”	“p65”	“CMV”	0.11
6.	“CASSLAGEQYF”	“HomoSapiens”	“p65”	“CMV”	0.2
7.	“CASSLNYEQYF”	“HomoSapiens”	“p65”	“CMV”	0.64
8.	“CASSLLGGDAETLYF”	“Mus-Musculus”	“M45”	“MCMV”	0.1

*Sequences 1–4 coincide features of stage-1 (see section 3.3), and sequences 5–8 coincide features found in stage-2. The last column is the IGoR probability (33)*.

## 4. Discussion

We have proposed a new way to look at TCR rep-seq data. By rebuilding the sequences into a network, and by following this network over temporal changes in the phenotype, we were able to identify changes in the repertoire that associate with changes in the phenotype. Using the proposed methodology, we demonstrated its utility in two different disciplines, namely, graph/network theory and statistical machine learning. Following a clustering process and further pruning, we generated a network for each sample/time-point. By summing up the sequences associated with the respective clusters measured at that time-point per sample, the nodes of each network represent the “activity” of the Clone-Attractors. We applied two graph measures on the networks: Betweenness-Centrality and Molecular-Topological-Index, and demonstrated its ability to discriminate the two populations, control and transgenic, with a rate of 0.8. The same Clone-Attractors were used for developing a two-stage classifier machine, separating control from transgenic, and further separating pre-cancer from cancer samples in the transgenic sub-population. This machine achieves an estimated true positive rate of 0.9 at a false positive rate of 0.1. A word of caution is in order here regarding the machine learning results at this time. As the amount of data available for the study was limited, it is reasonable to assume a certain level of over fitting, although this concern has been addressed by applying a robust estimation. Additional experimental data is required to further test our method.

This new way provides, in essence, a biologically-inspired means to perform dimensionality reduction on repertoire data. The Clone-Attractors are built using their biology, namely, their sequence similarities. When we collapse sequences onto the network representation, we use this biology to raise an alternative view of the system, in a different set of dimensions. However, this dimensionality reduction, as useful as it might be for data compression and representation, would not be interesting without exposing utility. Indeed, such utility is readily presented, by 1. stratifying different network behaviors in the two phenotypes we have studies: mice that develop tumor vs. mice that do not, and by 2. using the behavior of the Clone-Attractors to classify different samples according to their origin, as well as physiological state.

Further, we find that the CA themselves are associated with a number of curated sequences, that appear in context of a set of related and unrelated pheotypes, curated in the VDJdb database. This association, which may be interesting in and of itself, further provides context to the possible cognate peptides of the T cells. Since many of the TCR sequences identified in this manner (see Table 1) are associated with human and mouse viral peptides, the biology behind the association between these specific peptides and the tumor phenotype remains to be seen.

It is important to emphasize, however, that part of the public nature of many of the sequences is, in fact, an artifact of the measurement itself. The method used here is unable to provide a match between the alpha and beta sequences. In that case, a single beta sequence may actually represent a number of distinct T cell clones, which differ in their alpha sequence. In spite of this limitation, the conclusion of the computation used here, which is the success in classification, overcomes this issue and is able to deliver the reported results. It might be that with the progress in single-cell sequencing, we would be able to significantly improve over these classifications.

However, the *Clone-Attractor phase space* representation is more than merely a dimensionality reduction tool. We hypothesize that this space reflects the temporal status of the immune response to tumor progression as follows. CAs having small basin of attraction, i.e., that are composed of a small number of sequences, may be a normal immune response to antigens, pathogens, etc. This can be viewed as an extension of the clone notion. When the immune response fails to control those cells and the tumor evolves, it is possible that the immune system replicates further T cells with similar TCRs to explore the adjacent sequence space, resulting in a larger basin of Clone-Attractor. This CA is also expected to be more active as the tumor progress. As temporal data become more abundant, it might be possible to chart certain regions of the CA landscape and associate both dynamics and specific attractors with particular pathologies.

The work described here succesfuly stratifies two classes: mice that would devlop tumors and mice that would not. However, in the context of machine learning, these are also the only two classes included in the experimnet. That is, we do not know if the classification easily carries into the complexity of the heterogenetiy of human subjects. To be able to carry the method further, much research is still needed, both in animal models and in human samples. The actual span of relevant classes is not binary, but huge, and probably, since T cells are involved in most aspects of physiology, contains any phenotype in the physiology of organisms. To be able to achieve such resolutions, a larger set of data needs to combine over multiple experiments, to feed a much more informative model.

With continuous research into T Cell Repertoires, especially with recent progress in the ability to associate TCRs with specific peptides (34, 35), we expect many future studies to produce TCR repertoire data. These data may benefit from a network perspective such as the one proposed here. The example we provide here raises interesting questions regarding the biology behind Clone-Attractors in general and specifically in breast cancer. Our own research continues to follow these specific clones and their role in tumor progression. Other data sets may raise to the surface a novel set of clones. Combined, these efforts, the networks that they use and the attractor-network that they would build, may further promote our understanding of this complex phenomena.

## Author Contributions

AP conducted the analyses and wrote the manuscript. MG performed the original experiments, HP performed some of the analyses, data preparations and preprocessing. AZ performed experiments and designed experiments. AP, AZ, and SE conceived the studies and designed the experiments. AP and SE wrote the first draft of the manuscript, with input from all authors.

### Conflict of Interest Statement

The authors declare that the research was conducted in the absence of any commercial or financial relationships that could be construed as a potential conflict of interest.
