# Supplementary material for: Network Representation of T-Cell Repertoire— A Novel Tool to Analyze Immune Response to Cancer Formation
Source: Front Immunol. 2018 Dec 11;9:2913. doi: 10.3389/fimmu.2018.02913 (PMC6297828; doi:10.3389/fimmu.2018.02913)
Supplement: Supplementary file 1 [file Data_Sheet_1.PDF]

Supplementary Material: Network representation of T-Cell repertoire – A novel tool to analyze immune response to cancer formation

1 MAIN MATLAB CODE FUNCTIONS

The MATLAB functions and scripts below are given As-Is. At the time of submission, they are not part of a closed package and therefore no support will be given.

```
function  Analyze_CDR3_aa_repertoire()
% ==================================================
%
% This function/script assumes the following variables are available:
% aa_seq - cell array of CDR3 aa-sequences
% cdr3Lvl - a matrix of cdr3 level for each sequence at each time-point
% CGdb - data structure containing Control samples meta-data
% MGdb - data structure containing Transgenic samples meta-data
%
% Essentially, it is a wrapper for the Online clustering

seqLen = zeros(size(aa_seq));
for k=1:length(aa_seq);
    seqLen(k) = length(aa_seq{k});
end

% Remove extreme length  (most seqs are included (more than 96%))
idxRem = find(seqLen>19 | seqLen<7);
seqLen(idxRem) = [];
aa_seq(idxRem) = [];
beta_aa_seq(idxRem) = [];
cdr3Lvl(idxRem,:) = [];
cdr3Lvl_aa_beta(idxRem,:) = [];


idxC = 1:length(aa_seq);
[~, ii] = sort(seqLen(idxC),'ascend');  % Start clustering with the shorter
seqs
idxC = idxC(ii);
```

```
% Clustering params
scoreThr = 3;  % minimal score for in-cluster
weight_delete = 1;
weight_insert = 1;
weight_replace = 2;


if ~exist('clustM')
    clustM = [];
    clustM.params.scoreThr = scoreThr;
    clustM.params.weight_delete = weight_delete;
    clustM.params.weight_insert = weight_insert;
    clustM.params.weight_replace = weight_replace;

    minFarClustDist = 12;
    % Main call for online clustering
    [clustM, nDistCalc,MM] = clusterSeq(aa_seq, idxC,
     weight_delete,weight_insert,weight_replace, scoreThr, minFarClustDist);
    save clustRepDmat_aa MM clustM
    clear MM
end


cLen = [];
idx2Clust = zeros(size(seqLen));  % mapping seq# to cluster#
for m=1:length(clustM),
    cLen(m)=length(clustM(m).memIdx);
    idx2Clust(clustM(m).memIdx) = m;
end


% matrix of number of sequences per cluster per sample
seqInClustInSample=[];
actSeqInClustInSample=[];
nonactSeqInClustInSample=[];
for m = 1:size(cdr3Lvl,2)
    for ci = 1:length(clustM),
        seqInClustInSample(ci,m) = nansum(cdr3Lvl(clustM(ci).memIdx,m));
        actSeqInClustInSample(ci,m)=nansum(cdr3Lvl(clustM(ci).memIdx,m)>0);
        nonactSeqInClustInSample(ci,m) =
        nansum(cdr3Lvl(clustM(ci).memIdx,m)==0);
    end
end
```

```
% -----------------------------------------------------------------------------

function [clustM, nDistCalc, MM] = clusterSeq(NT_seq, idxC,
 weight_delete,weight_insert,weight_replace, scoreTh, minFarClustDist)
%
% Main online clustering function. Most expensive loop runs in parallel mode
%

% Initial memory allocation: assuming compression of at least 1:6
max_clustNum = round(length(idxC) / 6);

% Init
clustM(max_clustNum).rep = [];
clustM(max_clustNum).memIdx = [];
clustM(max_clustNum).ri = [];

clustNum = 1;
clustM(clustNum).rep = NT_seq{idxC(1)};
clustM(clustNum).memIdx = idxC(1);
clustM(clustNum).ri = 1;

%curRep = clustM(clustNum).rep;
nDistCalc = 0;

farClustTh = 3*scoreTh;  % Threshold to identify very far clusters;

% setting the distance matrix to "infinity"
clustRepDmat = 100*scoreTh*ones(max_clustNum,'uint8');
clustRepDmat(1,1) = 0;  % Distance Mat between cluster's centers
MM(1,1) = 0;

for k = 2:length(idxC)
    if mod(k,10000)==0
        disp([num2str(round(100*k/length(idxC))) '% ' num2str(clustNum)
        '  ' num2str(round(toc/60))])
    end

    found = 0;
    clustList = ones(clustNum,1);  % Relevant cluster list to consider

    for cj = 1:clustNum
        if ~clustList(cj)  % skip if cluster is not potentially relevant
        (i.e., too far from current seq)
            continue
```

```
        end
        score = edit_distance_weighted(clustM(cj).rep, NT_seq{idxC(k)},
         weight_delete,weight_insert,weight_replace);
        nDistCalc = nDistCalc + 1;
        % update clustList

        if clustNum > 2 && score > scoreTh &&  score < farClustTh-scoreTh+2
            ii = find(clustRepDmat(cj,1:clustNum) > max(minFarClustDist,
            score + scoreTh));  % farClustTh
            clustList(ii) = 0;
        end

        if score <= scoreTh
            clustM(cj).memIdx(end+1) = idxC(k);
            found = 1;
            % refine cluster representative by minimizing the SOD
            if length(clustM(cj).memIdx)==10 ||
            length(clustM(cj).memIdx)==20
            || length(clustM(cj).memIdx) == 50
                [clustM(cj).rep, ni] =
                 newRep_minSOD(NT_seq(clustM(cj).memIdx),weight_delete,
                 weight_insert,weight_replace);
                clustM(cj).ri(end+1) = ni;
            end
            break
        end
    end

    if found
        continue
    else  % create new cluster
        clustNum = clustNum + 1;
        clustM(clustNum).rep = NT_seq{idxC(k)};
        clustM(clustNum).ri = 1;
        clustM(clustNum).memIdx = idxC(k);
        % update cluster's dist. Mat
        tmpD = zeros(clustNum,1);
        if clustNum > 30
            cRep = clustM(clustNum).rep;
            parfor ci = 1:clustNum-1
                ciRep = clustM(ci).rep;
                tmpD(ci) = edit_distance_weighted(cRep, ciRep,
                 weight_delete,weight_insert,weight_replace);
            end
        else
```

```
            cRep = clustM(clustNum).rep;
            for ci=1:clustNum-1
                ciRep = clustM(ci).rep;
                tmpD(ci) = edit_distance_weighted(cRep, ciRep,
                 weight_delete,weight_insert,weight_replace);
            end
        end

        % assuming distances are positive and FAPP <100
        % This allocation is cheap
        if clustNum >= max_clustNum
            clustRepDmat = zeros(clustNum,'uint8');
        end
        clustRepDmat(1:clustNum,clustNum) = tmpD;
        clustRepDmat(clustNum,1:clustNum) = tmpD;
    end

end

clustM(clustNum+1:end) = [];


% ---------------------------------------------------------------------------
function [newRep,ni] = newRep_minSOD(clustSeq,weight_delete,weight_insert,
weight_replace)

seqNum = length(clustSeq);
sodMat = zeros(seqNum);

for si = 1:seqNum-1
    for sj = (si+1):seqNum
        sodMat(si,sj) = edit_distance_weighted(clustSeq{si}, clustSeq{sj},
         weight_delete,weight_insert,weight_replace);
    end
end
sodMat = sodMat + transpose(triu(sodMat));
[~,ni] = min(mean(sodMat) + 2*std(sodMat));
newRep = clustSeq{ni};

% ----------------------------------------------------------------------------

function score = edit_distance_weighted(s,t,weight_delete,weight_insert,
weight_replace)
  % EDIT_DISTANCE_WEIGHTED calculates a weighted string distance.
  % Weights (1,1,1) are the Levenshtein distance.
```

```
  %

  m=numel(s);
  n=numel(t);

  if nargin < 3, weight_delete=1; end
  if nargin < 4, weight_insert=1; end
  if nargin < 5, weight_replace=1; end

  d=zeros(m+1,n+1);

  % initialize distance matrix
  for i=1:m % deletion
    d(i+1,1)=d(i,1) + weight_delete;
  end
  for j=1:n % insertion
    d(1,j+1)=d(1,j) + weight_insert;
  end

  for j=2:n+1
      for i=2:m+1
          d1 = d(i-1,j-1);
          if s(i-1) ~= t(j-1)
              d1 = d1 + weight_replace;
          end
          d2 = d(i,j-1) + weight_delete;
          d3 = d(i-1,j) + weight_insert;
          tmp = min(d1, d2);
          d(i,j)=min(d3, tmp);
      end
  end

  score = d(m+1,n+1);

% ---------------------------------------------------------------------------
% ---------------------------------------------------------------------------

% Main function to analyze time-point data

function analyze_graph_dynamics()
%
% The script/function assumes the following variables are available:
% clustM - struct-array containing clusters calculated by 'clusterSeq'
% cdr3Lvl - the matrix of cdr3 level of each sequence at each time-point
% CGdb/MGdb - Control/Transgenic meta-data structures
```

```
% actSeqInClustInSample - matrix containing the number of sequences
% associated with each cluster
%

cLen = [];
for m=1:length(clustM),
    cLen(m)=length(clustM(m).memIdx);
end

% Rescaling each sample by its total number of copies
numCopies = sum(cdr3Lvl);

scaleF = zeros(size(cdr3Lvl,2),1);
CMedNC = []; for k=1:length(CGdb),
CMedNC(k) = median(numCopies(CGdb(k).idxInMat)); end
MMedNC = []; for k=1:length(MGdb),
MMedNC(k) = median(numCopies(MGdb(k).idxInMat)); end
for k=1:length(CGdb), scaleF(CGdb(k).idxInMat) =
CMedNC(k)./numCopies(CGdb(k).idxInMat); end
for k=1:length(MGdb), scaleF(MGdb(k).idxInMat) =
MMedNC(k)./numCopies(MGdb(k).idxInMat);
end

actSeqInClustInSample_scaled =
 actSeqInClustInSample.*repmat(scaleF(:).',size(actSeqInClustInSample,1),1);
actSeqInClustInSample_scaled = ceil(actSeqInClustInSample_scaled);

% Taking a subset of the clusters, such that each cluster chosen is
% represented in 'prc_control_tp' of the control time-points at least
minSeqInClust = 10;
prc_control_tp = 0.65;
interClustDist_th = 8;  % inter-cluster distance cutoff

idx1 = find(cLen>=minSeqInClust);
seqIC = actSeqInClustInSample_scaled(idx1,:);
idxCM = vertcat(CGdb.idxInMat);
idx2 = find(sum(seqIC(:,idxCM).'>0) >= prc_control_tp*length(idxCM));

idxRelevantClust = idx1(idx2);
clustM_r = clustM(idxRelevantClust);
clustRepDmat = zeros(length(clustM_r));

for ci=1:length(clustM_r)
    repi = clustM_r(ci).rep;
    parfor cj=(ci+1):length(clustM_r)
```

```
        repj = clustM_r(cj).rep;
        clustRepDmat(ci,cj) = edit_distance_weighted(repj, repi,
         weight_delete,weight_insert,weight_replace);
    end
end

% Distance matrix for Graph representation
clustDEgde = double(clustRepDmat);
if ~my_issymmetric(clustDEgde)
    clustDEgde = clustDEgde + clustDEgde';
end
actClustSamp = actSeqInClustInSample(idxRelevantClust,:);

% Get features for the graph in each time-point
% ============================
clustDEgde_th = clustDEgde;
clustDEgde_th(clustDEgde_th > interClustDist_th) = 0;
[graphFeat_C, comp_C] = calc_dbSamp_feat(CGdb,clustDEgde_th,actClustSamp);
[graphFeat_M, comp_M] = calc_dbSamp_feat(MGdb,clustDEgde_th,actClustSamp);

% =====================================================
% =====================================================

function [graphFeat, comp] =
calc_dbSamp_feat(curDB, clustAdjMat, seqInClustInSample)
%
% Calculate all features for a sample DB
%
% clustAdjMat - Distance matrix. '0' means no connection

% Some control parameters
minSubNet = 10; % minimal subnet size
numComponents = 5;  % number of largest components of a graph to compute
numMem = 10; % number of largest components to save

graphFeat = [];
comp = [];

for samp = 1:length(curDB)
    sDB = curDB(samp);
    tstart = tic;
    Feat = [];
    for ii=1:length(sDB.idxInMat)
        si = sDB.idxInMat(ii);
        [nComponents,sizes,members,~] = find_graphComponents(clustAdjMat,
```

```
            seqInClustInSample(:,si));
        comp(samp).tp(ii).members = members(1:min(numMem,length(members)));
        for j=1:min(numComponents,length(members))
            idx = members{j};
            if length(idx) < minSubNet, continue, end
            componentAdjMat = getSubAdjMat(idx, clustAdjMat);
            wtAdjMat = componentAdjMat;
            wtAdjMat(isnan(wtAdjMat)) = 0;
            wtAdjMat(wtAdjMat == inf) = 0;
            if j==1 % just the largest graph
                comp(samp).tp(ii).adjMat = wtAdjMat;
            end
            Feat(ii).comp(j) = getGraphFeatures(wtAdjMat);
        end
    end
    graphFeat(samp).Feat = Feat;
end

% ---------------------------------------------
function [graphFeat] = getGraphFeatures(adjMat)
%
% calc various graph features/measures
%

th_adjMat = adjMat;
th_adjMat(1:(size(adjMat,1)+1):end) = 1e6;
th_adjMat(th_adjMat==0) = 1e6; % zero edges are disconnected
th_adjMat = 1./th_adjMat;  % i.e., large weight is low connection

bwc = betweenness_centrality(sparse(th_adjMat));

clc = []; %closeness_centrality(adjMat);
%[bc,b,c] = bridging_centrality(adjMat);
bc = [];
mq1 = []; %modularity_n06(adjMat);
mq2 = []; %modularity_ws05(adjMat);
wid = within_module_degree(adjMat);
rc = []; %richclub_coefficient(adjMat,1);
swidx = []; %smallworldindex(adjMat);
ge = []; %graph_efficiency(adjMat);
nCyc = numCycles(adjMat);
[diam,rad,~,~] = my_graph_diameter(adjMat);
laplacSpect = graphLaplacSpect(adjMat);
```

```
% Calc features: AZI, HZ, MTI
if ~issparse(adjMat)
    adjMat=double(sparse(adjMat));
end

% We can take binary shortest path, or use weighted graph
% adjMat_bin = adjMat;
% adjMat_bin(adjMat_bin>0) = 1;
[sh_path]=all_shortest_paths(adjMat);

% Augmented zagreb Index , and Hyper-zagreb
azi = 0;
hz = 0;

deg = nansum(th_adjMat,1);

for i=1:length(deg)-1
    for j=i+1:length(deg)
        azpair = (deg(i)*deg(j)/(deg(i)+deg(j)-2))^3;
        if ~isinf(azpair)
            azi = azi + azpair;
        end
        hz = hz + (deg(i)+deg(j))^2;
    end
end

% molecular topological index
d_adjMat = full(adjMat);
fact = prctile(d_adjMat(:),99);
d_adjMat(d_adjMat==0) = Inf;
d_adjMat = fact*exp(-3/fact*d_adjMat);
d_adjMat(isinf(d_adjMat)) = 0;
sh_path(sh_path==0) = Inf;
sh_path = 2*fact./sh_path;
E = (d_adjMat+sh_path)*(deg+1e-5)';  % 1e-5 for disconnected nodes
mti = sum(E);

% pack all features
graphFeat.N = length(adjMat);
graphFeat.bwc = bwc;
graphFeat.clc = clc;
graphFeat.bc = bc;
graphFeat.mq1 = mq1;
graphFeat.mq2 = mq2;
graphFeat.wid = wid;
```

```
graphFeat.rc = rc;
graphFeat.swidx = swidx;
graphFeat.ge = ge;
graphFeat.nCyc = nCyc;
graphFeat.diam = diam;
graphFeat.rad = rad;
graphFeat.lapSpect = laplacSpect;
graphFeat.deg = deg;
graphFeat.azi = azi;
graphFeat.hz = hz;
graphFeat.mti = mti;


% ===================================================================
% ===================================================================
```

Main Machine-Learning Functions

```
function FC_Classification()
%-------------------------------------------------------

% SVM + CROSSVAL Classification based on FC
%
% Assuming the following variables are available:
% - actSeqInClustInSample
% - CGdb, MGdb
%
% Current version develops a 2-stage process: First classify
% between control/transgenic , then, within transgenic, classify
% precancer/cancer



useScaled = 0; % to use scaled data or original samples


% Taken from MGdb
idx_precanc = [MGdb(1).idxInMat(1:3); MGdb(2).idxInMat(1:2); ...
MGdb(3).idxInMat(1:3); MGdb(4).idxInMat(1:2); MGdb(5).idxInMat(1:5); ...
 MGdb(6).idxInMat(1:2); MGdb(7).idxInMat(1:2);  ...
    MGdb(8).idxInMat(1:2); MGdb(9).idxInMat(1:2); MGdb(10).idxInMat(1:4)];
idx_cancOnset = [MGdb(1).idxInMat(4); MGdb(2).idxInMat(3); ...
MGdb(3).idxInMat(4); MGdb(5).idxInMat(6); MGdb(6).idxInMat(3); ...
MGdb(8).idxInMat(3); MGdb(9).idxInMat(3); MGdb(10).idxInMat(5)];
```

```
idx_canc = [MGdb(1).idxInMat(5); MGdb(2).idxInMat(4:5); ...
MGdb(3).idxInMat(5:6); MGdb(4).idxInMat(3); MGdb(5).idxInMat(7:8);
MGdb(6).idxInMat(4:5); MGdb(7).idxInMat(3);  ...
    MGdb(8).idxInMat(4); MGdb(9).idxInMat(4); MGdb(10).idxInMat(6)];


idxTpC = [];
idxTpM = [];
for k=1:length(CGdb), idxTpC = [idxTpC; CGdb(k).idxInMat];end
for k=[1:3,4, 5:6, 7,8:10], idxTpM = [idxTpM; MGdb(k).idxInMat];end
idxTpAll = [idxTpC; idxTpM];

if useScaled
    idxComAll =
     find(sum(actSeqInClustInSample_scaled(:,idxTpAll)>0,2)>= ...
     length(idxTpAll)-3);
else
    idxComAll =
    find(sum(actSeqInClustInSample(:,idxTpAll)>0,2)>=length(idxTpAll)-3);
end

if useScaled
    featMatC = actSeqInClustInSample_scaled(idxComAll,idxTpC).';
    featMatPC = actSeqInClustInSample_scaled(idxComAll,idx_precanc).';
    featMatCO = actSeqInClustInSample_scaled(idxComAll,idx_cancOnset).';
    featMatCA = actSeqInClustInSample_scaled(idxComAll,idx_canc).';
else
    featMatC = actSeqInClustInSample(idxComAll,idxTpC).';
    featMatPC = actSeqInClustInSample(idxComAll,idx_precanc).';
    featMatCO = actSeqInClustInSample(idxComAll,idx_cancOnset).';
    featMatCA = actSeqInClustInSample(idxComAll,idx_canc).';
end

featMatComb = [featMatC; featMatPC; featMatCO; featMatCA];

% ==============================================
%  CREATE 2-CLASSES (control / trans-noCancer + trans-onset + trans-cancer
% ==============================================

clas = [1*ones(length(idxTpC),1); 2*ones(length(idx_precanc),1); ...
 2*ones(length(idx_cancOnset),1); 2*ones(length(idx_canc),1)];
costMat = [0 1; 1.0 0];
costS.ClassNames = [1 2];

costS.ClassificationCosts = costMat;
```

```
% ===== start with 2 Features, select best combination(s),
% then try to add '1' feature at a time

numFeat = 2;
featSet2 = nchoosek(1:size(featMatComb,2),numFeat);
lossMat = [];
confMat2 = [];
t0=tic;
for k=1:size(featSet2,1)
    dat = log(featMatComb(:,featSet2(k,:)));
    dat(isinf(dat)) = 0;
    SVMModel =  fitcsvm(dat,clas,'KernelFunction','gaussian', ...
    'KernelScale','auto','BoxConstraint',5,'Cost',costMat);
    CVSVMModel = crossval(SVMModel);
    lossMat(k,:) = kfoldLoss(CVSVMModel,'Mode','individual');
end

bestPairs = find(mean(lossMat.')<0.26);  % Empirically chosen

boxConst = 10;
bestK = 50;
maxFeat = 10;

% Generating best bottom-up feature selection
[featSet_allD, lossMat_allD, models_all] = ...
botUpFeatSelection(featSet2(bestPairs,:), featMatComb, clas, ...
lossMat(bestPairs,:), boxConst, costMat, bestK, maxFeat);

% Test robustness of best models by adding noise
% ===========================================
numMach = 10; % number of best classifiers to use
nD = 5; % features dimention (actually nD+1 since starting from pairs
nsLvl = 0.2; % noise std added
numTrials = 50;

params = [];
params.boxConst = boxConst;
params.nsLvl = nsLvl;
params.numMach = numMach;
params.numTrials = numTrials;
nsLvlDel = 0.05;  % noise level jump

predM = []; % predM dimensions (samples,trials,machines)
tic,
```

```
for nD=1:length(featSet_allD)
    for nsV=1:9
        nsLvl = (nsV-1)*nsLvlDel;
        params.nsLvl = nsLvl;
        predM(nD,nsV).res = ...
        evalModel_robust(featMatComb, clas, featSet_allD{nD}, params );
        predM(nD,nsV).params = params;
        [nD nsV round(toc)]
    end
end

% ROC curve
% =========

vMach = 1:10;
aucM = [];
xROC = [];
yROC = [];
for nD=1:length(featSet_allD)
    for nsV=1:9
        y1=[]; auc1 = [];
        for k=1:size(predM(nD,nsV).res,2)
            % The '-' is because perfcurve assumes the first class has
            %results equll to '1'
            % avg over machines
            [x1,y1(k,:),~,auc1(k)] = ...
            perfcurve(clas,-mean(predM(nD,nsV).res(:,k,vMach),3),1, ...
            'xVals',[0:0.01:0.25 0.3:0.05:1],'UseNearest','off');
        end
        xROC(nD,nsV,:) = x1;
        yROC(nD,nsV,:)  = mean(y1);
        aucM(nD,nsV) = mean(auc1);
    end
end

figure,hold on,grid
for nD=1:size(yROC,1)
    plot(squeeze(xROC(nD,1,:)),squeeze(yROC(nD,1,:)),'-'),
end

% ====================================================================
% Using direct train/test data sets
prcTrain = 0.8;
params = [];
params.boxConst = boxConst;
```

```
params.prcTrain = prcTrain;
params.numMach = numMach;
params.numTrials = 200;
predMt = []; % predM dimensions (samples,trials,machines)
tic,
for nD=1:length(featSet_allD)
    [predMt(nD).res, predMt(nD).trueClas] = evalModel_test(featMatComb, ...
    clas, featSet_allD{nD}, params );
    predMt(nD).params = params;
    [nD round(toc)]
end

vMach = 1:10;
aucMt = [];
xROCt = [];
yROCt = [];
for nD=1:length(featSet_allD)
    y1=[]; auc1 = [];
    for k=1:size(predMt(nD).res,2)
        tClas = squeeze(predMt(nD).trueClas(:,k,vMach(1)));
        if length(unique(tClas)) == 1
            y1(k,1:length(tClas)) = nan;
            auc1(k) = nan;
            continue
        end
        [x1,y1(k,:),~,auc1(k)] = ...
        perfcurve(tClas,-mean(predMt(nD).res(:,k,vMach),3),1,...
        'xVals',[0:0.01:0.25 0.3:0.05:1],'UseNearest','off');
    end
    xROCt(nD,:) = x1;
    yROCt(nD,:)  = nanmean(y1);
    aucMt(nD) = nanmean(auc1);
end



% ======================================================================
% ======================================================================
% Stage-2: Classify precancer from cancer for Transgenic.
% Assuming the Transgenic samples has been separated from the Control
% ======================================================================

clas2 = [1*ones(length(idx_precanc),1); 2*ones(length(idx_canc),1)];
idxTr = [idx_precanc; idx_canc];
```

```
featMat2 = featMatComb(idxTr,:);

numFeat = 2;
featSet2C = nchoosek(1:size(featMat2,2),numFeat);
lossMatC = [];
costMat = [0 1; 1 0];

for k=1:size(featSet2C,1)
    dat = log(featMat2(:,featSet2C(k,:)));
    dat(isinf(dat)) = 0;
    SVMModel =
    fitcsvm(dat,clas2,'KernelFunction','gaussian','KernelScale','auto',...
    'BoxConstraint',5,'Cost',costMat);
    CVSVMModel = crossval(SVMModel);
    lossMatC(k,:) = kfoldLoss(CVSVMModel,'Mode','individual');
end

bestPairsC = find(mean(lossMatC.')<0.26); % Empirically chosen

boxConst = 10;
bestK = 30;
maxFeat = 10;

% Generating best bottom-up feature selection
[featSetC_allD, lossMatC_allD, modelsC_all] = ...
botUpFeatSelection(featSet2C(bestPairsC,:), featMat2, clas2, ...
lossMatC(bestPairsC,:), boxConst, costMat, bestK, maxFeat);

% Performance evaluation
numMach = 10; % number of best classifiers to use
nD = 5; % features dimention (actually nD+1 since starting from pairs
nsLvl = 0.2; % noise std added
numTrials = 50;

params = [];
params.boxConst = boxConst;
params.nsLvl = nsLvl;
params.numMach = numMach;
params.numTrials = numTrials;
nsLvlDel = 0.05;  % noise level jump

predMC = []; % predM dimensions (samples,trials,machines)
tic,
for nD=1:length(featSetC_allD)
    for nsV=1:6
```

```
        nsLvl = (nsV-1)*nsLvlDel;
        params.nsLvl = nsLvl;
        predMC(nD,nsV).res = evalModel_robust(featMat2, clas2, ...
        featSetC_allD{nD}, params );
        predMC(nD,nsV).params = params;
        [nD nsV round(toc)]
    end
end

vMach = 1:10;
aucM2 = [];
xROC2 = [];
yROC2 = [];
for nD=1:size(predMC,1)
    for nsV=1:size(predMC,2)
        y1=[]; auc1 = [];
        for k=1:size(predMC(nD,nsV).res,2)
            resk = mean(squeeze(predMC(nD,nsV).res(:,k,vMach)),2);
            [x1,y1(k,:),~,auc1(k)] = perfcurve(clas2,-resk,1,'xVals',...
            [0:0.01:0.25 0.3:0.05:1],'UseNearest','off'); %avg over machines
        end
        xROC2(nD,nsV,:) = x1;
        yROC2(nD,nsV,:)  = mean(y1);
        aucM2(nD,nsV) = mean(auc1);
    end
end


% ======================================================================
% Using direct train/test data sets
prcTrain = 0.7;
params = [];
params.boxConst = boxConst;
params.prcTrain = prcTrain;
params.numMach = numMach;
params.numTrials = 200;
predMt2 = []; % predM dimensions (samples,trials,machines)
tic,
for nD=1:length(featSet_allD)
    [predMt2(nD).res, predMt2(nD).trueClas] = evalModel_test(featMat2,...
     clas2, featSetC_allD{nD}, params );
    predMt2(nD).params = params;
    [nD round(toc)]
end
```

```
vMach = 1:10;
aucMt2 = [];
xROCt2 = [];
yROCt2 = [];
for nD=1:length(featSetC_allD)
    y1=[]; auc1 = [];
    for k=1:size(predMt2(nD).res,2)
        tClas = squeeze(predMt2(nD).trueClas(:,k,vMach(1)));
        if length(unique(tClas)) == 1
            y1(k,1:length(tClas)) = nan;
            auc1(k) = nan;
            continue
        end
        [x1,y1(k,:),~,auc1(k)] = ...
        perfcurve(tClas,-mean(predMt2(nD).res(:,k,vMach),3),1,'xVals',...
        [0:0.01:0.25 0.3:0.05:1],'UseNearest','off');
    end
    xROCt2(nD,:) = x1;
    yROCt2(nD,:)  = nanmean(y1);
    aucMt2(nD) = nanmean(auc1);
end

%==========================================================================

function [featSet_allD, lossMat_allD, models_all] = ...
botUpFeatSelection(featPairs, featMat, clas, lossMat_0, ...
boxConst, costMat, bestK, maxFeat)
%
% starting from best pairs, add one feature at a time and evaluate
% choose bestK(e.g. 100) from current dimension and continue till no
% improvement is achieved, or maxFeat of features

featSet = featPairs;

[lossVal_0, idx_0] = sort(mean(lossMat_0'));  % get best pairs

lossMat_allD = [];
featSet_allD = [];
featSet_allD{1} = featSet;
lossMat_allD{1} = lossVal_0;

models_all = [];

tic,
```

```
numFeat = 3; % Assuming we started with pairs
cnvg = 0;
bestPerf = mean(lossVal_0(1:10));
iter = 2; % First is for pairs

% iterations over number (dimension) of features
while ~cnvg && numFeat <= maxFeat
    featSetComb = [];
    for k=1:size(featSet,1)
        for m = 1:size(featMat,2)
            if length(unique([featSet(k,:) m])) < numFeat, continue, end
            featSetComb = [featSetComb; [featSet(k,:) m]];
        end
    end
    featSetComb = unique(sort(featSetComb,2),'rows');
    cLossMat = [];
    cModels = [];
    for k=1:size(featSetComb,1)
        cFeat = featSetComb(k,:);
        dat = log(featMat(:,cFeat));
        dat(isinf(dat)) = 0;
        SVMModel =  fitcsvm(dat,clas,'KernelFunction','gaussian',...
        'KernelScale','auto','BoxConstraint',boxConst,'Cost',costMat);
        CVSVMModel = crossval(SVMModel,'Leaveout','on');
        cModels{k} = CVSVMModel;
        cLossMat(k,:) = kfoldLoss(CVSVMModel,'Mode','individual');
    end

    % find best models and get new features set
    [lossVal, idx] = sort(mean(cLossMat'));
    featSet = featSetComb(idx(1:bestK),:);

    featSet_allD{iter} = featSet;
    lossMat_allD{iter} = lossVal;
    models_all{iter} = cModels{idx(1:bestK)};
    if mean(lossVal(1:10)) > bestPerf
        cnvg = 1;
    else
        bestPerf = mean(lossVal(1:10));
    end
    disp('********************************')
    disp([numFeat, bestPerf*100])
    disp('********************************')

    numFeat = numFeat + 1;
```

```
    iter = iter + 1;
end

%==================================================================================
function predM = evalModel_robust(datMat, clas, featSet, params )
%
% Assuming binary classifier

boxConst = params.boxConst;
nsLvl = params.nsLvl;
numMach = params.numMach;
numTrials = params.numTrials;

predM = [];

for mm=1:numMach
    dat = log(datMat(:,featSet(mm,:)));
    dat(isinf(dat)) = 0;

    SVMModel1 = fitcsvm(dat,clas,'KernelFunction','gaussian',...
    'KernelScale','auto','Leaveout','on','BoxConstraint',boxConst);

    % Random noise repeats
    for tr = 1:numTrials
        pdat = dat + nsLvl*randn(size(dat));
        pdat(pdat<0) = 0;  % no negative values

        perf=[];
        perfPa=[];
        predV = [];
        for k=1:length(SVMModel1.Trained)
            CompactSVMModel1 = SVMModel1.Trained{k};
            teIdx = find(SVMModel1.Partition.test(k));
            [~,scorePred1] = predict(CompactSVMModel1,pdat(teIdx,:));
            predV(teIdx) = scorePred1(:,2);
        end

        predM(:,tr,mm) = predV;
    end
end

%==================================================================================

function [predM,trueClas] = evalModel_test(datMat, clas, featSet, params )
%
```

```
% Assuming binary classifier

boxConst = params.boxConst;
numMach = params.numMach;
numTrials = params.numTrials;
prcTrain = params.prcTrain; % percentage for test
numTrain = round(prcTrain*length(clas));

predM = [];
trueClas = [];

% Random repeats
for tr = 1:numTrials
    rr = randperm(length(clas));
    idx = rr(1:numTrain);
    idxTest = rr(numTrain+1:end);

    for mm=1:numMach

        dat = log(datMat(:,featSet(mm,:)));
        dat(isinf(dat)) = 0;
        datTrain = dat(idx,:);
        datTest = dat(idxTest,:);
        outTest = clas(idxTest);

        SVMModel1 = fitcsvm(datTrain,clas(idx),'KernelFunction',...
        'gaussian','KernelScale','auto','CrossVal','off',...
        'BoxConstraint',boxConst);

        [~,scorePred1] = predict(SVMModel1,datTest);
        predM(:,tr,mm) = scorePred1(:,2);
        trueClas(:,tr,mm) = outTest;
        %(outTest*2-3) transform to binary (-1,1)
    end
end


%==================================================================
```

2 SUPPLEMENTARY TABLES AND FIGURES

2.1 List of Sequences Used As Features

The following table S1 provides the list of sequences used for the classification process. As described in the **Methods** section, these sequences are the centers of the Clone-Attractors (CA). Those CA's where chosen such that they are found in most time-points.

1. CASSPREQYF	2. CASNYEQYF	3. CASSLAEQYF	4. CTCSDTQYF
5. CASSLETLYF	6. CASSRKDTQYF	7. CTCSAFEQYF	8. CASSLSTQYF
9. CASSSQNTLYF	10. CTCSGTYEQYF	11. CASSYNSDYTF	12. CTCSAETLYF
13. CASSQEYEQYF	14. CASSGGTEVFF	15. CASSLDTEVFF	16. CTCSADNTEVFF
17. CASLGGYEQYF	18. CASRQNTEVFF	19. CASSGTGEQYF	20. CASSLGGNTLYF
21. CASSSSAETLYF	22. CTCSAGAEQFF	23. CASLNQDTQYF	24. CASRGQYEQYF
25. CASSPRYAEQFF	26. CASSPTDTQYF	27. CASSPGLYEQYF	28. CASSLEGDTQYF
29. CTCSAGGTEVFF	30. CTCSAGNERLFF	31. CASSSYNSPLYF	32. CASSRGNSPLYF
33. CASRTNTGQLYF	34. CTCSAGGNTLYF	35. CASSQQYAEQFF	36. CASSHGQDTQYF
37. CASSGTTNSDYTF	38. CASSLDNYEQYF	39. CASSLGRDTQYF	40. CTCSAAQDTQYF
41. CASSLDQQDTQYF	42. CTCSAPSAETLYF	43. CASSQDTGQLYF	44. CASSIDRGEQYF
45. CASSQATGQLYF	46. CASSQDGDEQYF	47. CSSRDRQDTQYF	48. CTCSAENYAEQFF
49. CTCSADNTGQLYF	50. CASSPGGSDYTF	51. CASSRGTGQLYF	52. CASSLGPNSDYTF
53. CASSQQGNTEVFF	54. CASSRTDQDTQYF	55. CASLANTGQLYF	56. CASSQTNQDTQYF
57. CASSLRANTEVFF	58. CASSRNNQDTQYF	59. CASSLTVSYEQYF	60. CASSLSGNTEVFF
61. CASSGAYAEQFF	62. CASSSNNYAEQFF	63. CASSPTGGYEQYF	64. CASSQAGGYEQYF
65. CASSLGQSYEQYF	66. CASRLGVQDTQYF	67. CASSLGGFAEQFF	68. CASSLVNYAEQFF
69. CASSLRGSYEQYF	70. CASSLSPTGQLYF	71. CASSQDWAQDTQYF	72. CASSWTGEDTQYF
73. CASKLGGQDTQYF	74. CASSDWGWDTQYF	75. CASSDSSGNTLYF	76. CASSLGTSAETLYF
77. CASSLSGSQNTLYF	78. CASSPGLGDTQYF	79. CASSQDGSQDTQYF	80. CASSLLGGAETLYF
81. CTCSAAGSQNTLYF	82. CASSAGGNYAEQFF	83. CASSLGRNTGQLYF	84. CASSPGGGQNTLYF
85. CASSPGANTGQLYF			

Table S1. List of sequences used for classification

2.2 Figures

The table shown in Figure S1 is taken from (Gordin et al., 2018).

REFERENCES

Gordin, M., Philip, H., Zilberberg, A., Cohen Gidon, M., Margalit, R., Clouser, C., et al. (2018). Mice developing mammary tumors evolve t cell sequences shared with human breast cancer patients. *bioRxiv* doi:10.1101/371260

Sample_id	# cells	Raw reads	Alpha			Beta			group
			Copies	Unique NT sequences	Unique AA sequences	Copies	Unique NT sequences	Unique AA sequences	
A_2	32064	723712	85327	1263	1219	232198	3003	2938	control_young
A_4	27964	535959	106943	3464	3168	185003	5476	5230	control_young
A_7	106503	730212	99261	8816	7622	227057	19443	17787	control_old
A_9	29695	637360	104969	678	671	240207	1366	1346	control_old
B_3	126011	647621	-	-	-	243750	27155	24013	control_young
B_4	126711	581011	-	-	-	226716	35432	30938	control_young
B_6	26085	568208	86848	1246	1215	172698	2339	2304	control_old
B_8	59652	576945	80271	2384	2246	200162	5186	5009	control_old
C_2	123170	651732	66430	753	736	212338	2083	2059	control_young
C_4	86484	746969	-	-	-	271564	27814	24538	control_young
C_5	103905	923329	385040	11272	10516	385040	11272	10516	control_young
C_6	22735	802049	116365	989	972	230074	1841	1814	control_old
C_7	83428	346810	46161	2534	2369	116751	6213	5907	control_old
C_8	15584	1017191	85478	284	282	275614	641	637	control_old
C_9	15584	523715	89757	658	650	171295	1144	1136	control_old
D_2	128461	421400	-	-	-	149785	2376	2338	control_young
D_4	79088	543240	95016	2346	2187	218530	4631	4481	control_young
D_5	10182	600106	68041	201	200	174093	528	525	control_young
D_6	43027	830358	130438	1975	1887	272778	4015	3895	control_old
D_7	96447	1083692	171337	10396	8842	340475	19975	18157	control_old
D_8	11906	581158	90907	768	755	189112	1462	1446	control_old
E_4	39288	507655	90476	2321	2162	189405	4270	4118	control_young
E_6	36585	1073086	154155	2080	1998	379892	4435	4333	control_old
E_8	13602	543944	87329	1251	1215	201248	2532	2491	control_old
E_9	116084	583740	67526	1043	1011	206646	2960	2908	control_old
F_2	46966	475816	59923	2903	2668	169413	7058	6683	no_cancer
F_3	46966	2312834	231515	879	865	756883	2683	2643	no_cancer
F_4	131442	528731	40967	5596	4986	208806	18533	16913	no_cancer
F_5	129099	692292	120926	7963	6973	225972	14483	13458	pre_cancer
F_7	216697	739307	120341	9164	7901	273558	19190	17526	cancer
G_2	17043	484586	64057	938	910	139881	2002	1964	no_cancer
G_3	83739	490738	-	-	-	173201	12197	11246	no_cancer
G_5	144398	595335	-	-	-	169581	9030	8540	pre_cancer
G_6	69022	799409	80974	932	918	183392	2090	2065	cancer
G_7	79109	816287	104596	17011	13780	249379	39320	34222	cancer
H_2	38453	567175	79845	4369	3935	195858	8722	8192	no_cancer
H_3	50269	524514	99457	4787	4254	198872	8423	7903	no_cancer
H_4	124606	447490	83153	5941	5237	168351	10852	10048	no_cancer
H_6	7034	949643	134941	355	354	234373	653	649	pre_cancer
H_7	10176	495208	76313	317	316	154007	626	623	cancer
H_8	152151	544731	75481	6329	5622	184779	14986	13808	cancer
I_4	193660	300981	49950	11832	9995	124731	28330	25162	no_cancer
I_5	96363	781927	134555	6712	5945	272228	12963	12066	no_cancer
I_6	28989	636704	95469	1581	1514	214157	3341	3256	pre_cancer
J_2	144614	572782	49086	1825	1710	145251	4731	4520	no_cancer
J_3	26329	1015608	148475	758	746	375973	1819	1802	no_cancer
J_4	91338	529691	90156	5491	4866	191619	10561	9851	no_cancer
J_5	77937	745616	140542	3931	3617	213861	6004	5777	no_cancer
J_6	45291	542297	74374	1912	1806	169588	4272	4147	no_cancer
J_7	34020	661602	109047	2187	2092	226989	4336	4206	pre_cancer
J_8	36790	1708688	212218	1186	1154	463049	2490	2457	cancer
J_9	34984	400204	64135	710	698	127169	1257	1242	cancer
K_2	190167	1437881	74511	440	438	148237	771	769	no_cancer
K_4	57570	434631	71424	1435	1358	164672	2998	2928	no_cancer
K_5	107631	416509	63021	2264	2157	128584	4494	4327	no_cancer
K_6	17492	492313	76982	1212	1191	175489	2954	2906	cancer
K_7	131795	432717	49687	1141	1104	106906	2403	2358	cancer
L_2	23572	596425	74605	9486	8067	228171	24582	21884	no_cancer
L_5	102133	696688	107780	4394	4022	231699	9063	8584	no_cancer
L_6	12365	588921	104142	1003	982	187046	1731	1717	pre_cancer
M_2	42588	573640	-	-	-	219794	16317	14823	no_cancer
M_4	133728	461569	80092	11267	9482	181396	24049	21542	no_cancer
M_6	16257	687307	102585	1257	1229	236790	2843	2803	pre_cancer
M_7	112515	778042	-	-	-	255111	13674	12777	cancer
N_2	74616	720944	74694	2096	1968	209818	4890	4717	no_cancer
N_3	84879	653289	98991	3821	3515	211537	7791	7419	no_cancer
N_5	44765	556754	102816	1823	1746	177587	3048	2987	no_cancer
N_7	92808	677063	110246	8058	7132	246097	16984	15645	cancer
O_2	77757	610413	-	-	-	205298	21875	19585	no_cancer
O_4	126723	558054	91567	9278	7878	220692	20251	18332	no_cancer
O_5	92134	738591	121411	5171	4683	232310	9479	8960	no_cancer
O_6	37852	735687	109656	3015	2817	228839	5863	5653	no_cancer
O_7	124562	705997	-	-	-	242723	19064	17431	pre_cancer
O_8	195153	527388	74832	15493	12691	196024	37787	32846	cancer

Figure S1. Usable count of sequences per sample and time-point.
